# The Role of *Candida albicans SPT20* in Filamentation, Biofilm Formation and Pathogenesis

**DOI:** 10.1371/journal.pone.0094468

**Published:** 2014-04-14

**Authors:** Xiaojiang Tan, Beth Burgwyn Fuchs, Yan Wang, Weiping Chen, Grace J. Yuen, Rosalyn B. Chen, Elamparithi Jayamani, Cleo Anastassopoulou, Read Pukkila-Worley, Jeffrey J. Coleman, Eleftherios Mylonakis

**Affiliations:** 1 Department of Respiration, Overseas Medical Center, Nanfang Hospital, Southern Medical University, Guangzhou, P. R. China; 2 Department of Medicine, Infectious Diseases Division, Rhode Island Hospital, Warren Alpert Medical School of Brown University, Providence, Rhode Island, United States of America; 3 Division of Infectious Diseases, Massachusetts General Hospital, Boston, Massachusetts, United States of America; 4 School of Pharmacy, Second Military Medical University, Shanghai, P. R. China; 5 Department of Molecular Biology, Massachusetts General Hospital, Harvard Medical School, Boston, Massachusetts, United States of America; 6 Program in Immunology, Harvard Medical School, Boston, Massachusetts, United States of America; 7 Phillips Academy, Andover, Massachusetts, United States of America; Université de Nice-CNRS, FRANCE

## Abstract

*Candida albicans* is a ubiquitous fungus, which can cause very serious and sometimes life-threatening infections in susceptible patients. We used *Caenorhabditis elegans* as a model host to screen a library of *C. albicans* mutants for decreased virulence and identified *SPT20* as important for virulence. The transcription co-activator *SPT20* was identified originally as a suppressor of Ty and solo δ insertion mutations, which can cause transcription defects in *Saccharomyces cerevisiae*. It is resistant to the toxicity caused by overexpression of GAL4-VP16. We constructed a *C. albicans spt20Δ/Δ* mutant and found the *spt20Δ/Δ* strain was significantly less virulent than the wild-type strain SC5314 in *C. elegans* (*p* < 0.0001), *Galleria mellonella* (*p* < 0.01) and mice (*p* < 0.001). Morphologically, *spt20Δ/Δ* mutant cells demonstrated a “snow-flake” shape and clustered together; prolonged culture times resulted in increased size of the cluster. The clustered morphology was associated with defects in nuclei distribution, as the nuclei were not observed in many cellular compartments. In addition, the *C. albicans spt20Δ/Δ* mutant resulted in defects in hyphae and biofilm formation (compared to the wild-type strain, *p* < 0.05), and sensitivity to cell wall and osmotic stressors, and to antifungal agents. Thus our study demonstrated a role of *C. albicans SPT20* in overall morphology and distribution of nuclear material, which may cause the defects in filamentation and biofilm formation directly when this gene is deleted.

## Introduction

*Candida albicans* is part of the human flora that can be isolated from the gastrointestinal tract, vagina, and mouth in healthy individuals. Nevertheless, when the anatomical barrier is damaged or when host immunity declines then an invasive fungal infection can follow [1]. *C. albicans* is the fourth most common pathogen isolated from blood cultures and candidemia is associated with mortality rates as high as 35% [2] or even higher [3], [4]. A characteristic of *C. albicans* is that it can make the switch between several morphological forms: budding yeast, pseudohyphae, and true hyphae, according to the environmental conditions [5], [6]. The pathogenic ability of *C. albicans* is closely related to the change between these morphological forms [7], [8]. Additionally, the formation of hyphae [9] and biofilms [10], the integrity of the cell wall [11], the rapid adaptive capacity to external environmental condition [12] all contribute to virulence and are all related to morphogenesis.

Here we report *SPT20* is involved in *C. albicans* virulence and is essential for hyphal and biofilm formation. The transcription co-activator *SPT20* encodes a 604 amino acid nuclear protein that is rich in glutamine and asparagines [13] and was identified originally as a suppressor of Ty and solo δ insertion mutations, which can cause transcription defects in *Saccharomyces cerevisiae* [14]. There is ample evidence that *S. cerevisiae SPT20* mutants have some broader transcriptional defects when compared with the other *SPT* genes [13]–[15]. Genome-wide expression analysis showed that *SPT20* controls about 10% of normal transcription in *S. cerevisiae* [16]. Of note is that *SPT20* has also been identified by another name, *ADA5*, as it is a member of the *ADA* group of genes, and it is resistant to the toxicity caused by overexpression of GAL4-VP16 which blocks the transcription factor combined with transcription complexes, thus killing the yeast cells [13]. Interestingly, we found that in *C. albicans*, *SPT20* is associated with cell morphology; *spt20Δ/Δ* cells cluster together and demonstrate a “snow-flake” shape. Deformity in cell morphology is accompanied by defects in nuclear localization.

## Materials and Methods

### Strains and growth conditions

*C. albicans* strains were grown in YPD (1% yeast extract, 2% peptone, and 2% dextrose) at 30°C. Nourseothricin-sensitive clones were identified by their small colony size and confirmed by re-streaking on YPD agar plates containing 200 µg/mL nourseothricin (Werner Bioagents, Jena, Germany) as described previously [17].

For the nematode studies, the *C. elegans glp-4*; *sek-1* strain was propagated on nematode growth medium on lawns of *Escherichia coli* OP50 using standard methods[18].

### Generation of a homozygous mutant and re-integrated strain

The flipper cassette from pSFS2 containing the dominant nourseothricin resistance marker *CaSAT1* was used to generate the *spt20Δ/Δ* mutant [17]. This *SAT1* flipping strategy was used to avoid any interference from the auxotrophic selective marker genes in our virulence studies. The DNA fragment for homologous recombination was generated as follows: PCR was used to amplify a 924 bp *Apa*I-*Xho*I DNA fragment containing an upstream region and the 5′ end of *SPT20* gene with primers *SPT20*-up-FWD and *SPT20*-up-RV (**Table S1**). The fragment was then cloned into the pSFS2 vector upstream of the *MAL2p-CaFLP-CaSAT1* cassette, yielding plasmid pSFS2-*SPT20*up. A 726 bp *Sac*II-*Sac*I fragment containing a down-stream region and the 3′ end end of the *SPT20* gene was amplified by PCR with primers *SPT20*-down-FWD and *SPT20*-down-RV (**Table S1**) and cloned into the pSFS2-*SPT20*up vector downstream of the *MAL2p-CaFLP-CaSAT1* cassette using the *Sac*II and *Sac*I restriction sites, yielding the final plasmid pSFS2-*SPT20*updwn. The pSFS2-*SPT20*updwn plasmid was digested with *Apa*I and *Sac*I, and the 5856 bp fragment was incorporated into *C. albicans* SC5314 using the lithium acetate method [19]. Nourseothricin-resistant transformants were evaluated for the appropriate insert location by PCR (**Table S2**), and the correct transformants were used for further work. After excising the nourseothricin resistance marker (*caSAT1*), the same method was used to delete the remaining functional allele of *SPT20*. Another round of integration/excision yielded *SPT20* homozygous mutant *spt20Δ/Δ*. The primers used to identify all the strains are listed in **Table S2**.

To re-integrate *SPT20* into the *SPT20* null mutant, *spt20Δ/Δ*, a 3640 bp *Apa*I-*Xho*I fragment containing the complete open reading frame as well as 790 bp of upstream and 594 bp of downstream flanking sequences was amplified with the primers *SPT20*-FWD and *SPT20*-RV (**Table S1**) and substituted for upstream and 5′ end of *SPT20* gene sequence in pSFS2-*SPT20*updwn at the *Apa*I and *Xho*I restriction site to generate the plasmid pSFS2-*SPT20*Rec. The pSFS2-*SPT20*Rec plasmid was digested with *Apa*I and *Sac*I, and the 7848 bp fragment was used to reintroduce the *SPT20* sequence into the *C. albicans* mutant *spt20Δ/Δ*. Nourseothricin-sensitive derivatives were obtained in the following process, yielding the *SPT20* reconstituted strain *spt20Δ/SPT20* for further investigations. The primers used to identify the strains are listed in **Table S2**. We were unable to produce a strain that re-integrated the *SPT20* sequence at the second allele site. However, testing of the single loci re-integrated strain exhibited morphology and virulence comparable to the wild-type strain.

### In vitro hyphal formation and biofilm growth assays

For hyphal formation, *C. albicans* strains were incubated with 1 mL serum for 60 min at 37°C with agitation in the dark, then collected by centrifugation and washed twice with phosphate buffered saline (PBS). Cells were suspended in Spider media and incubated at 37°C for 20 h [18], then photographed using an Olympus Bx51 series upright microscope. For biofilm growth evaluation [20], strains of *C. albicans* were grown in YPD medium overnight at 30°C, diluted to an OD_600_ of 0.5 in 2 mL Spider medium, and added to a sterile 12-well plate containing a prepared silicone square measuring 1.5 × 1.5 cm (cut from silicone sheets Bentec Medical Inc., Woodland, CA) that had been pretreated overnight with bovine serum (Sigma-Aldrich). The inoculated 12-well plate was incubated with gentle agitation (150 rpm) for 90 min at 37°C for adhesion to occur. The squares were washed with 2 mL of PBS to remove any un-adhered cells, and moved to a fresh 12-well plate containing 2 mL of fresh Spider medium. This plate was incubated at 37°C for an additional 60 h with agitation (150 rpm) to allow for biofilm formation. The silicone platform and attached biofilm were removed from the wells, dried overnight, and weighed the following day. The total biomass of each biofilm was calculated by subtracting the weight of the silicone platform material prior to biofilm growth from the weight after the drying period and adjusting for the weight of a control pad exposed to no cells. The average total biomass for each strain was calculated from four independent samples. Statistical significance was determined by the analyses of variance (ANOVA). For comparison between two *C. albicans* isolates, the Student *t* test was used. A p-value of less than 0.05 was considered significant.

### Sensitivity assays

The sensitivity to cell stressors was evaluated using the cell wall stress agent sodium dodecyl sulfate (SDS) and osmotic stress agent sodium chloride (NaCl). *C. albicans* strains were grown overnight and then suspended in YPD at 2×10^6^ cells/mL. Ten fold serial dilutions from 10^4^ to 10^1^ of the wild-type, *spt20Δ/Δ* and *spt20Δ/SPT20* re-integrated strains were spotted in a volume of 5 µL on YPD agar plates with the indicated chemical agent added. Cells were grown under 25°C, 30°C, and 37°C conditions until colonies appeared.

### Virulence assay using the C. elegans infection model

The assay was carried out using an established protocol [18]. In brief, freshly grown *C. albicans* cells were inoculated into 2 mL of YPD broth and allowed to grow overnight at 30°C. The following day, 100 µL of yeast was spread into a square lawn on a 10-cm plate containing brain heart infusion (BHI) agar and kanamycin (45 µg/mL), followed by incubation for approximately 20 h at 30°C. Synchronized adult *C. elegans glp-4*; *sek-1* nematodes grown at 25°C were carefully washed from plates containing their normal food source (*E. coli* OP50 strain) using sterile M9 buffer. Approximately 400 to 500 washed worms were then added to the center of the *C. albicans* lawns. The plates were incubated at 25°C for 4 h. Worms were then carefully moved into a 15 mL conical tube and washed four times with sterile M9. Sixty worms were then transferred into a single well of a six-well tissue culture plate (Corning, Inc.) containing 2 mL of liquid medium (80% M9, 20% BHI) and kanamycin (45 µg/mL). Worms were scored daily and dead worms were removed from the assay. Microscopy of nematodes was performed by using Nomarski optics on a Zeiss AxioImager microscope.

To screen for mutations that affect *C. albicans* virulence, we evaluated a miscellaneous homozygous mutant library from the Fungal Genetics Stock Center (Kansas City, MO), deposited by Dr. Aaron Mitchell. Worms were infected as described above and scored for visibility of fungal filaments protruding through the worms cuticle and survival at 72 h post infection. Survival was determined by the ability to respond to touch with a platinum wire. The control group was infected with strain DAY286, the parent of the mutant strains in the library collection.

### Virulence assay using the Galleria mellonella infection model

Inocula were prepared by growing *C. albicans* strains at 30°C overnight with agitation. Cells were collected with centrifugation and washed 3 times with PBS. Yeast cells were counted using a hemocytometer. *G. mellonella* larvae (Vanderhorst Wholesale Inc., St. Marys, OH) at the final instar stage were inoculated with 10^6^ cfu of *C. albicans* suspended in PBS. Each infection group contained 16 randomly chosen larvae of the appropriate weight (330 ± 25 mg). The inoculum was injected in a 10 µL volume directly to the last left, pro-leg using a Hamilton syringe [21]. After inoculation, larvae were incubated at 37°C. A mock inoculation with PBS was included as a control for each experiment to observe for killing due to physical trauma or infection by pathogenic contaminants. The number of dead larvae was scored daily.

### Virulence assay using a murine candidiasis model

A disseminated murine candidiasis model [22] was selected for the assay. Strains SC5314, *spt20Δ/Δ*, *spt20Δ/SPT20* were used to infect mice. Cultures were grown overnight then washed 3 times with PBS. CD-1, six-week old, female mice were infected with 1.5 × 10^6^ CFUs suspended in PBS via a tail vein injection in a 100 µl volume. Twelve mice were inoculated per strain tested and observed daily. Animal survival was evaluated by using the Kaplan-Meier method and differences were determined by using the log-rank test (STATA 6; STATA, College Station, TX). A *p* value of < 0.05 was considered statistically significant.

Fungal burden in the kidneys was assessed using four mice per infection strain [23]. We harvested the kidneys from mice aseptically 2 days post infection. Tissues were weighed and homogenized in sterile PBS by use of a Tissue Tearor (model 398; Biospec Products Inc., Racine, WI). Serial dilutions were plated on YPD agar plates containing 100 µg/mL ampicillin, 100 µg/mL streptomycin, and 45 µg/mL kanamycin. The cfu/g kidney were counted after growth at 30°C for 48 h. Statistical analyses were performed using ANOVA and post hoc (Bonferroni and Student–Newman–Keuls) tests.

### Fungal cells staining with periodic acid Schiff (PAS)

Histopathological analysis was performed to assess kidney infestation. The murine candidiasis model was prepared as described above. Two days after inoculation, kidneys were removed aseptically from each mouse before being fixed in 10% neutral buffered formalin. Kidneys were embedded in paraffin and sections were stained with Periodic Acid Schiff (PAS) to reveal the hyphal structure of the fungal pathogens [23].

### Staining of nuclei

Cells were stained for nuclei according to the protocol by Kopecka *et al*. and Fuchs *et al*. [24], [25]. The strains were grown in YPD overnight at 30°C. Nuclei were identified by staining with 0.1 µg/mL DAPI for 15 min. The stained cells were collected then washed twice with 1% BSA in PBS for 5 min followed by a five minute rinse in 0.1% BSA in PBS. Cells were suspended in Vectashield (Vector Laboratories, Inc.) and visualized with an Olympus microscope. All observations were confirmed with 3 independent cell cultures.

### Fixation and staining of Calcofluor White

Cells were fixed by adding 40% Formaldehyde (50 µL) to 450 µL cell cultures for 30 min and washed 2 times with PBS. Subsequently, cells were re-suspended in Calcofluor solution (Sigma) for 5 min at room temperature and washed 5 times with PBS. Then the stained cells were re-suspended in 10 µL mounting medium and observed using an Olympus confocal microscope.

### Drug susceptibility tests

For the susceptibility assays, we grew cultures in YPD at 30°C with agitation. Cells were diluted in PBS to a density of 2.0×10^6^ cells/mL. The strains were plated as ten fold serial dilutions, spotting 5 µL of each dilution onto YPD agar plates supplemented with fluconazole (FLC; 2.5 µg/mL), amphotericin B (AMB; 1 µg/mL), 5-fluorocytosine (5FC; 4 µg/mL) or caspofungin (CAS; 0.075 µg/mL) and onto drug-free YPD agar plates as a control. Plates were incubated for 48 h at 35°C.

### Ethics statement

All experiments were approved by Massachusetts General Hospital Research Animal Care Subcommittee and as outlined in the Guide for the Care and Use of Laboratory Animals of National Institutes of Health.

## Results

Using a *C. elegans* infection model, we screened a *C. albicans* mutant library that consisted of 86 strains, for mutants that exhibited decreased virulence compared to strain DAY286. Through the course of our screening assay, we identified the *SPT20* mutant strain as causing significantly less death in the *C. elegans* worms than the control strain DAY286. More specifically, the control infection produced an average of 47.6% dead worms with visible filaments protruding through the worm cuticle with a SD of 10.3%.However, the worms infected with the *spt20* mutant strain did not produce any dead worms with protruding filaments. *SPT20* deletion strains were developed for further analysis in order to confirm the role of this gene and the contributions of *SPT20* in *C. albicans* virulence.

### The *spt20Δ/Δ* mutant has reduced virulence in the *C. elegans* and the *G. mellonella* infection models

**Figure 1:**
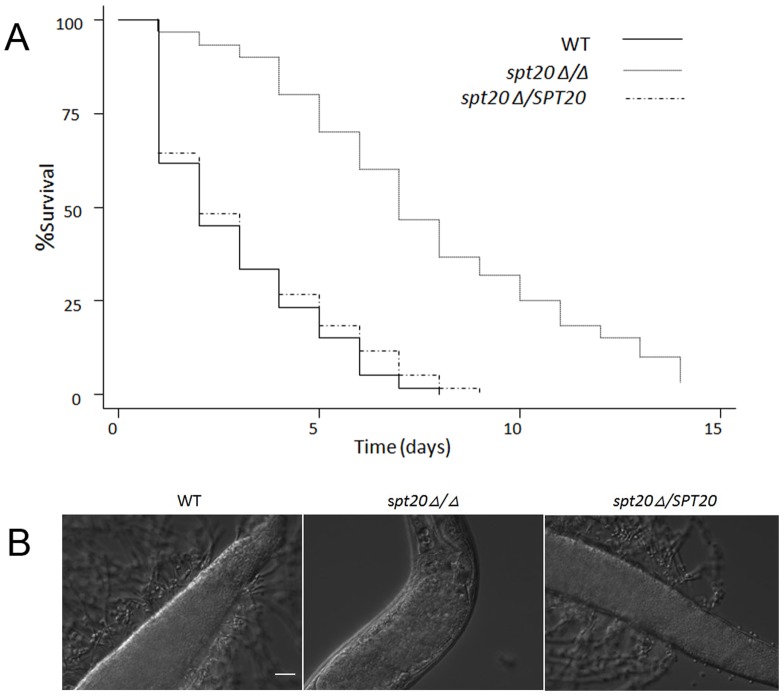
*SPT20* is essential for *C. albicans* virulence in the *C. elegans* infection model. (A) Survival over 120 h (n  =  60 worms infected per strain of *C. albicans*). Compared to infection with *C. albicans* SC5314, the *spt20Δ/Δ* mutant was avirulent to *C. elegans* (*p* < 0.0001). The reintegration of *SPT20* restored the virulence of *C. albicans* to the wild-type level. (B) The images show *C. elegans glp-4*;*sek-1* nematodes infected by *C. albicans* strains SC5314, *spt20Δ/Δ* and *spt20Δ/SPT20* for 4 h and then moved into pathogen-free liquid media. On day 4 of the assay, the wild-type and *spt20Δ/SPT20* infected worms exhibited a lethal infection with hyphae penetrating the worm cuticle. The hyphae were absent in the *spt20Δ/Δ* infected worms. The scale bar in Fig.1B represents 20 µm.

We confirmed the impact of *SPT20* deletion in virulence using the *C. elegans* infection model (**Figure 1**). In this series of experiments, we performed the nematode assays in more detail with daily scoring of dead/alive nematodes. We found that more than half of the worms infected with the wild-type SC5314 (or the re-integrated strain *spt20Δ/SPT20*) died within the first 48 h after infection (**Figure 1A**) and almost half the dead worms had visible hyphae piercing the cuticle (**Figure 1B**). At 120 h, >80% of the worms infected with the wild-type strain SC5314 or the re-integrated strain *spt20Δ/SPT20* were dead, while only 30% of the worms were dead in the *spt20Δ/Δ* infection group (**Figure 1A**; *p* < 0.0001 compared to the wild-type strain or the reintegrated strain). Furthermore, no hyphae were observed in the *spt20Δ/Δ* infection group (**Figure 1B**).

**Figure 2:**
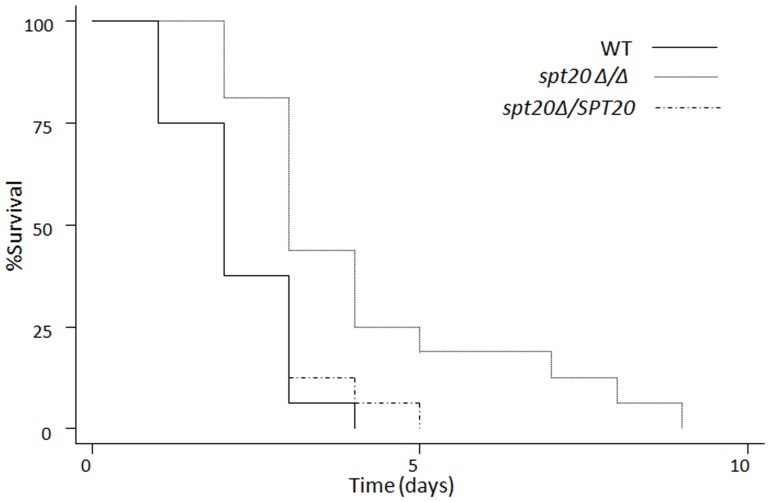
*SPT20* influences virulence of *C. albicans* in the *G. mellonella* infection model. Survival over 216 h (9 days) at 37°C (n  =  16 larvae per strain of *C. albicans*). *p* < 0.01 compared to the groups infected with the wild-type or the *spt20Δ/SPT20* reintegrated strain.

We confirmed the decreased pathogenicity of the *spt20Δ/Δ* strain in an insect model. In the *G. mellonella* infection model, the *spt20Δ/Δ* was significantly less virulent than the wild-type strain SC5314 at 37°C (**Figure 2**; *p* < 0.01). All the larvae in the wild-type strain died within 4 days and the re-integrated strain group died within 5 days while the *spt20Δ/Δ* infection group died within 9 days (**Figure 2**; *p* < 0.01).

### The *spt20Δ/Δ* mutant has attenuated virulence in the murine candidiasis model

**Figure 3:**
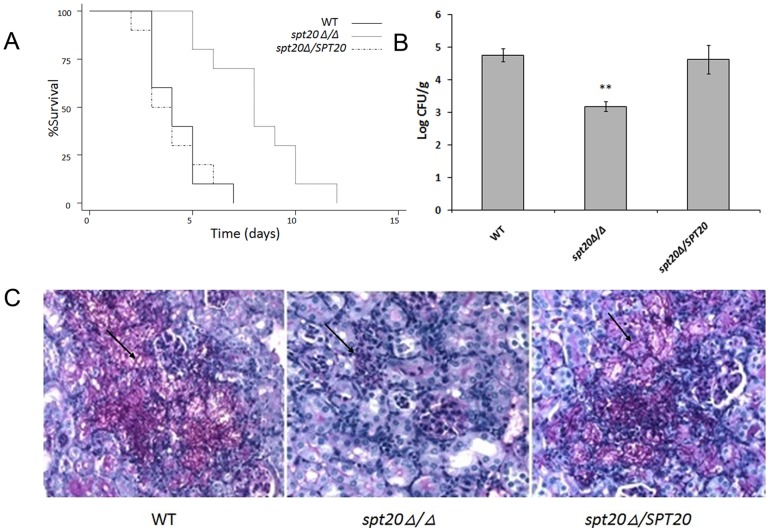
*SPT20* is required for virulence of *C. albicans* in the murine candidiasis model. Mice were inoculated via the tail vein with 1.5 × 10^6^ cfu of the indicated strains of *C. albicans*. (A) Survival over 21 days (n  =  12 mice per strain of *C. albicans*). *p* < 0.001 for *spt20Δ/Δ* strain compared to either the wild-type or *spt20Δ/SPT20* reintegrated strain. (B) Fungal burden of the kidneys of mice infected with the wild-type, *spt20Δ/Δ* and *spt20Δ/SPT20* reintegrated strain two days after inoculation (n  =  4 mice per strain of *C. albicans*). *p < 0.01 for *spt20Δ/Δ* strain compared to either the wild-type or *spt20Δ/SPT20* re-integrated strain. (C) Thin sections of kidneys were stained with PAS after 2 days of infection. Arrows indicate *C. albicans* filaments in the tissues.

The *spt20Δ/Δ* strain was significantly less virulent than the wild-type strain SC5314 (*p* < 0.001) (**Figure 3A**). More specifically, all 12 mice inoculated with the wild-type strains within 7 days post infection by tail vein injection, while only 4 out of 12 mice in the *spt20Δ/Δ* group were dead at the same period.

We also evaluated the fungal burden in the mouse kidneys as part of the pathogenicity assessment. The fungal burden in the *spt20Δ/Δ* group was significantly lower than that in the wild-type SC5314 infection group (*p* < 0.01; **Figure 3B**). Evaluation of the kidney tissue by histopathology illustrated the differences in the burden and morphology of the infecting fungi. PAS staining revealed a large number of fungi in the kidneys of mice infected with the wild-type or the re-integrated strains while rare fungal cells were observed in the kidneys from the mice infected with the *spt20Δ/Δ* strain (**Figure 3C**). We also observed differences in the morphological state of the infecting fungi. Hyphae were visible in the tissues of the wild-type and re-integrated strains infected mice but not in the kidney of mice infected with the *spt20Δ/Δ* strain (**Figure 3C**). Of note is that the fungal burden of the kidneys of mice infected with *spt20Δ/SPT20* re-integrated strain was restored, and the P value was not significantly different compared to the wild type.

### The role of *SPT20* in *C. albicans* cell wall integrity

**Figure 4:**
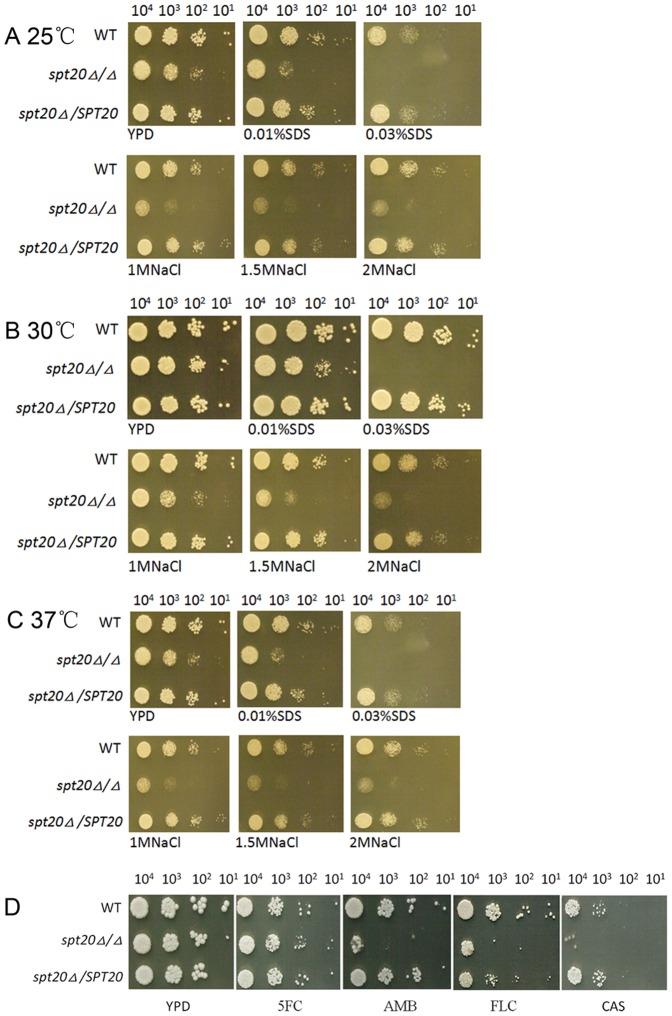
The disruption of *SPT20* affected *C. albicans* stress responses and antifungal susceptibility. Ten-fold serial dilutions of the wild-type, *spt20Δ/Δ*, and *spt20*/*SPT20* were evaluated for susceptibility to cell wall stressors and antifungal agents. The *spt20Δ/Δ* mutant was specifically susceptible to SDS and NaCl at all the temperatures tested, 25°C (A), 30°C (B) and 37°C(C). The *spt20Δ/Δ* mutant was hypersensitive to amphotericin B, fluconazole and caspofungin, but was not sensitive to 5-fluorocytosine (D).

To examine the cell wall integrity, we tested the susceptibility of wild-type strain SC5314, the *spt20Δ/Δ* mutant and the re-integrated strain *spt20Δ/SPT20* to the cell wall stress agent SDS and osmotic stress agent NaCl. Interestingly, the *spt20Δ/Δ* mutant was susceptible to 0.01%, 0.03% SDS and 1 M,1.5 M, 2 M NaCl at all the temperatures tested, 25°C, 30°C and 37°C (**Figure 4A, 4B and 4C**). Thus, the disruption of *SPT20* affected *C. albicans* stress responses and suggests that *SPT20* plays a role in maintaining cell wall integrity.

### Sensitivity to antifungal agents

In our drug susceptibility testing, the wild-type, *spt20Δ/Δ* and *spt20Δ/SPT20* re-integrated cells grew similarly on YPD agar plates and on YPD agar plates supplemented with 5FC, whereas the *spt20Δ/Δ* mutant was hypersensitive to amphotericin B, fluconazole and caspofungin compared to the wild-type and *spt20Δ/SPT20* re-integrated strains (**Figure 4D**).

### *SPT20* is involved in *C. albicans* morphology

**Figure 5:**
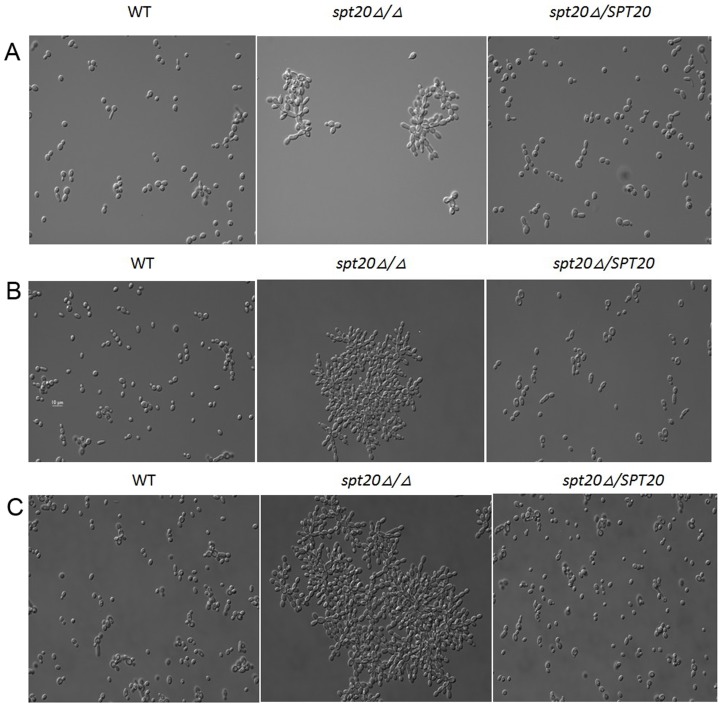
Micrographs of *C. albicans* morphology. Cell morphology was examined. Micrographs after 2(A), 8 h (B), and 24 h (C) of growth at 30°C are presented for the wild-type, *spt20Δ/Δ* and *spt20Δ/SPT20* reintegrated strain. The wild-type and *spt20Δ/SPT20* re-integrated cells separate and appear to have a round morphology at all 3 time points while *spt20Δ/Δ* cells cluster together and demonstrate a “snow-flake” shape.

An examination of the cell morphology at 30°C growth temperature was established. *C. albicans spt20Δ/Δ* has a morphological defect (**Figure 5**). Micrographs of *C. albicans* morphology after 2, 8, and 24 h of growth at 30°C are presented. We found that the *spt20Δ/Δ* cells cluster together and demonstrate a “snow-flake”shape rather than the normal separated and round shape of the wild-type and *spt20Δ/SPT20* re-integrated strain. The longer the cells are cultured, the larger the cell cluster.

**Figure 6:**
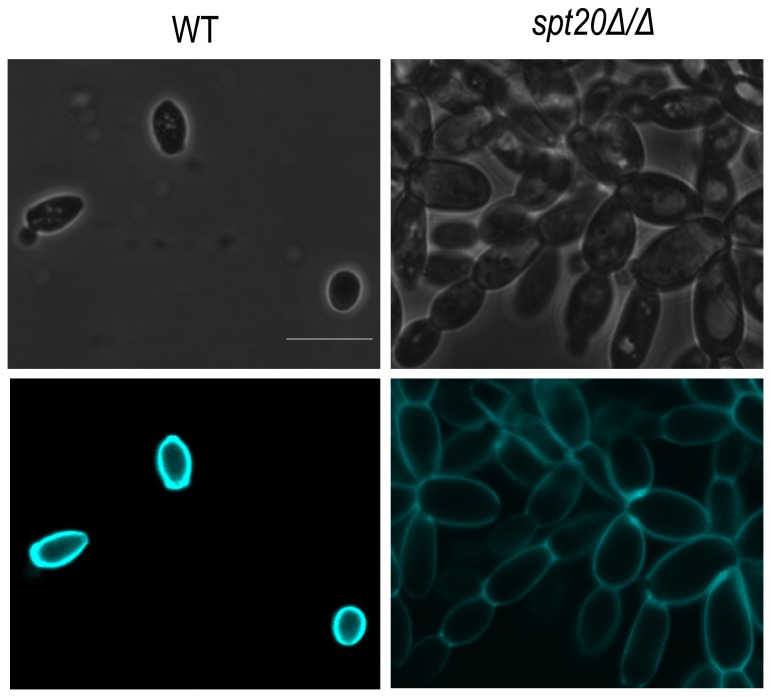
Calcofluor White staining of the wild-type and *spt20Δ/Δ*. Calcofluor White specifically stains chitin. *Spt20Δ/Δ* did not exhibit defects in chitin distribution. Bar  =  5 µm.

To further validate the clustering morphology, we evaluated the cells for defect in chitin. We analyzed chitin distribution in wild-type strain and *spt20Δ/Δ* strain using Calcofluor white and tested the expression of *CHT3* by RT-PCR. The results showed that *spt20Δ/Δ* did not exhibit defects in chitin distribution (**Figure 6**) and the expression of *CHT3* between wild-type strain and *spt20Δ/Δ* strain have no statistical differences (**Figure S1**).

### *SPT20* is associated with hyphal and biofilm formation

**Figure 7:**
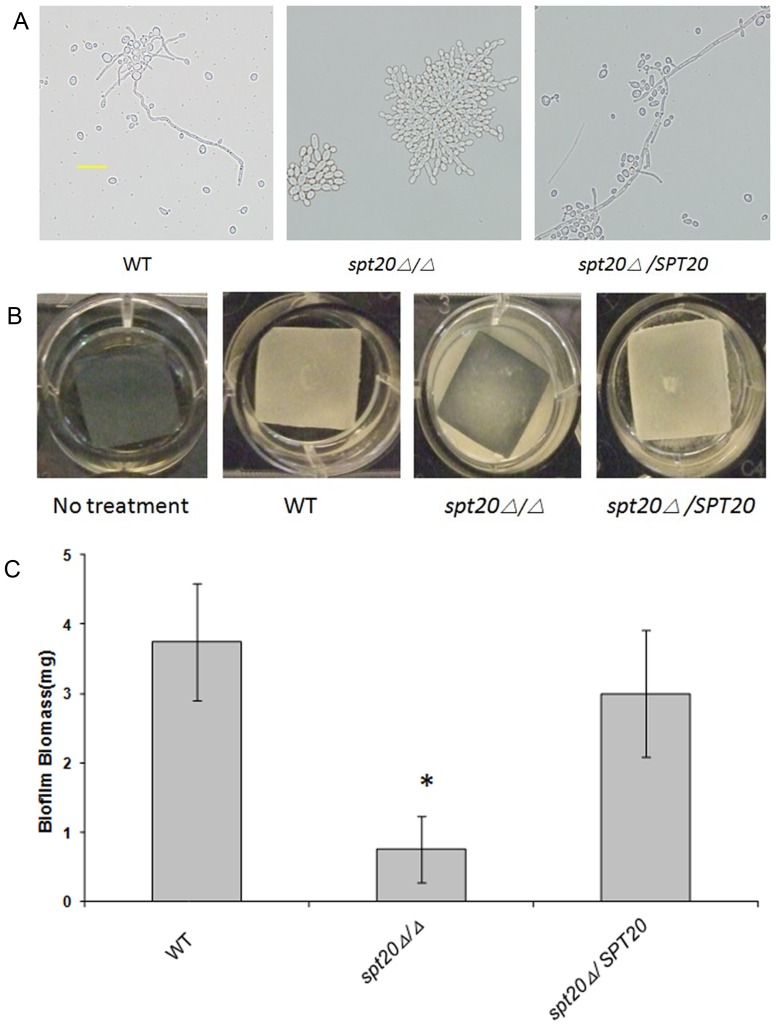
Hyphae and biofilm defects of the *spt20Δ/Δ*. (A) *C. albicans* strains SC5314, *spt20Δ/Δ* and *spt20Δ/SPT20* were grown in Spider medium for 20 h at 37°C and examined at ×400 magnification. Compared to the *C. albicans* SC5314, *spt20Δ/Δ* showed attenuated hyphae formation *in vitro* (also showed attenuated hyphal formation in vivo, see **Figure 1**). This phenotype was restored in the *spt20Δ/SPT20* reintegrated strain. The scale bar given in panel A represents 20 µm. (B) Strains were grown under *in vitro* biofilm assay conditions for 60 h. Biofilm formed on the silicone surface of the SC5314 and *spt20Δ/SPT20* strains but failed to form on silicone sheets seeded with *spt20Δ/Δ*. (C) Dry weight of the biofilm biomass. Standard deviations are depicted and based on 6–8 silicone pad measurements. *p < 0.05.

The visible difference in hyphae formation observed in the *C. elegans* infection assay and the lack of filaments in the infected kidney tissue, suggests that *SPT20* is needed for hyphae formation, and plays an important role in biofilm formation. We confirmed this role by studying hyphal development and biofilm formation *in vitro*. The results showed that the *spt20Δ/Δ* mutant was defective in hyphal (**Figure 7A**) and biofilm formation (*p* < 0.05; **Figure 7B and 7C**) compared to either the wild-type strain or the re-integrated strain.

### Defects in nuclear localization

**Figure 8:**
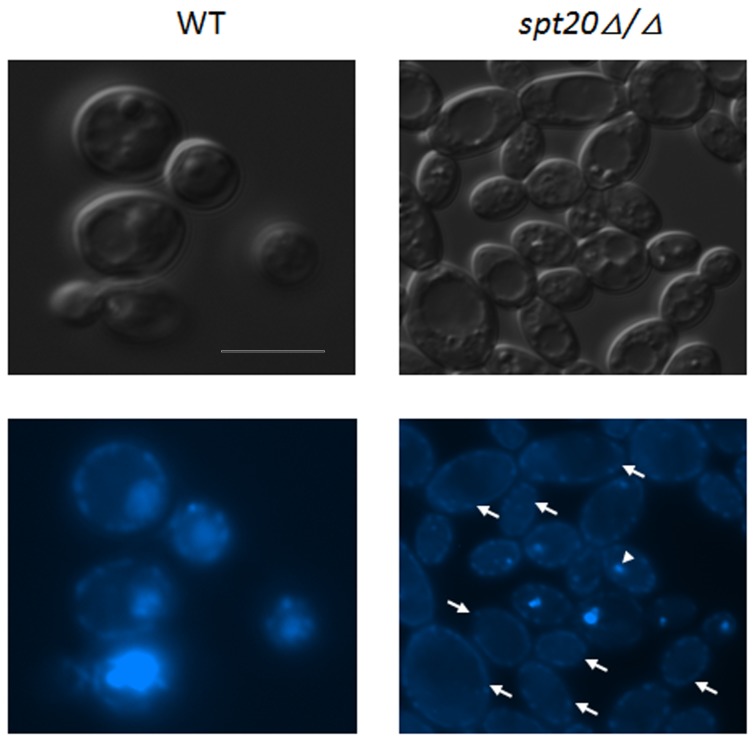
Nuclear defects in the *spt20Δ/Δ* cells. The light field image on the upper right shows a cluster of cells. The image on the lower right is a DAPI staining of the same cell cluster complex. The arrowhead indicates the location of the nucleus. Arrows indicate the cell complexes that lack a nucleus. Bar  =  5 µm.

In the“snow-flake”morphology, clustered cells appear to have defects in the ability of the daughter cells to separate from the mother cells, thus causing the clustering phenotype. We investigated whether the cell complexes all have nuclei using 6-diamidino-2-phenylindole dihydrochloride (DAPI) to evaluate the presence of nuclei in the complexes of the clustered cells. Interestingly, many gathered cells show defects in the location of nuclei (**Figure 8**). This indicates that the nucleus is not properly dividing or being distributed to the dividing cells. Overall, the lack of nuclei in some gathered compartments indicates a role for *SPT20* in nuclear division. The lack of nuclear material in complexes of the clustered cells may also be associated with the cytokinesis between the cell complexes of the clustered *SPT20* mutant cells.

## Discussion

In this report, we show that in *C. albicans*, *SPT20* plays a role in maintaining cell wall integrity. The defects in the cell wall are coupled with altered morphology and the inability of *spt20Δ/Δ* cells to form hyphae and generate a biofilm. Strikingly, these defects translate into reduced pathogenicity of the strain in multiple infection models.

The defects associated with cell morphology are characterized by a clustering effect, suggesting that *spt20Δ/Δ* cells are defective for cytokinesis, the mother and daughter cells do not separate properly. Moreover, some of the *spt20Δ/Δ* cells lacked nuclei therefore it is more accurate to describe some of the cells as “compartments” or “complexes”. These clusters are able to enlarge in the number of compartments that comprise the overall entity over time through the continuation of cell proliferation without complete separation. Interestingly, the *SPT20* mutant daughter cells of *S. cerevisiae* do not separate well from the mother cell [26], but the cells appears to have a nucleus [27], an aspect that differs in the *C. albicans* clustered complexes.

In *S. cerevisiae*, the growth rate of the deletion mutant is slow, and the *SPT20* mutant displays an elongated and irregularly bud morphology instead of the normal round morphology of wild-type cells [14], [28]. Although both fungi display morphological defects they differ in the rate of growth. Our examination of growth on solid media indicated that the *SPT20* mutant strain grew at rates comparable to the wild-type strain.

Adapting to variations in the environment is a basic feature for microorganism survival and, through adaptive evolutionary mechanisms, this process is under strict regulation and control. In *C. albicans*, we found that *SPT20* is involved with the ability of the fungal cells to adapt to compounds such as SDS that are toxic to the cell wall as well as to high osmolarity caused by sodium chloride. Similarly, in *S. cerevisiae SPT20* is essential for cell survival during environmental stress conditions [26], [29]. It's worth noting that *SPT20* maintains the structural integrity and the function of SAGA complex[30]–[33]. *SPT20* and SAGA complex in *S. cerevisiae* and *C. albicans* are also highly conserved [29]. In *S. cerevisiae*, *HOG1* signal transduction pathways can regulate intracellular synthesis and storage of glycerol, which resulted in increased intracellular osmotic pressure to improve the adaption to high osmolarity environments [34], [35]. SAGA is necessary for cell survival at hyperosmotic conditions [36]. In the hyperosmotic environment, it activates the HOG1 MAP kinase and is transferred from the cytoplasm to the nucleus, phosphorylating Sko1, and forms the Sko1-Cyc8-Tup1-Hog complex that recruits SAGA to the modified chromosome so that the RNA polymerase II binding to the promoter to initiate transcription [36].

Cell wall sensitivity to stress appears to be a conserved attribute between *SPT20* of *S. cerevisiae* and *C. albicans*. In *S. cerevisiae*, *SPT20* is hypersensitive to caspofungin [37], a drug that targets the fungal glucan synthase, important for the cell wall. Amphotericin B and azole target (directly or indirectly) the cell membrane and not the cell wall. In *C. albicans*, Vandeputte and colleagues found that *SPT20* the *C. albicans* mutant is more susceptible to 3 antifungal agents: amphotericin B, fluconazole and caspofungin than the wild-type strain, no alteration in sensitivity to flucytosine, a drug that targets the nuclear membrane [38]. This finding prompted us to confirm the *spt20Δ/Δ* mutation sensitivity to the antifungal agents using our generated strain. As shown in Figure 4D, our results were consistent with what was report by Vandeputte *et al*. The *spt20Δ/Δ* strain was more susceptible to compounds that targeted that cell membrane or wall than nuclear targeted 5FC. This suggests that the *spt20Δ/Δ* membrane defects are specifically associated with the cell wall or cell membrane and not to membranes in general and does not affect the nuclear membrane.

In *S. cerevisiae*, *SPT20* is believed to be required for the overall structural integrity and function of SAGA, a complex that facilitates the binding of TATA-binding proteins, because there is no intact SAGA complex in *SPT20* deletion mutants[39]-[42].SAGA controls 10% of all adjustable genes, and is known to be stress inducible [43]. Researches have shown that the SAGA complex of *C. albicans* is similar to the SAGA complex of *S. cerevisiae* [26]. Environmental stress conditions that influence gene expression that tends to be SAGA dominated include: heat, oxidation, acidity and nitrogen starvation, conditions that also influence the cell wall integrity pathway. In *S. cerevisiae*, SAGA regulates expression of *FKS1* and *ERG11*, involved in cell wall glucan synthase and ergosterol synthesis, respectively [43].

An important part of *C. albicans*' pathogenicity is its ability to form hyphae and generate biofilm, which play important roles in the colonization of medical devices. The hyphal form belongs to the overall factor of *C. albicans*' virulence by invading epithelial and endothelial cells and evading phagocytes that cause the release of hydrolytic enzymes, forming biofilms that colonize medical devices, and lead to tissue damage. This process requires active changes to the cell wall in order to accommodate expanding volume, relying on actin to maintain the cytoskeletal structure during the process of hyphae and pseudohyphae formation. Our investigation of *Spt20* suggests that it plays a role in biofilm formation and filamentation. The *spt20Δ/Δ* showed attenuated hyphal formation within the clustered morphology. The defective filaments were unable to support the formation of a biofilm, as assessed by the reduced biofilm mass. Importantly, the inability to form hyphae was reflected in the pathogenicity studies by the inability to produce hyphae that could penetrate the worm cuticle in the *C. elegans* infection model or the lack of hyphae penetration the kidney tissue in the murine model. The aspect of pathogenicity is a differing attribute between *S. cerevisiae* and *C. albicans*, where in the former does not cause disease but the latter can be an opportunistic pathogen. Thus the role that *SPT20* plays in *C. albicans* can contribute to virulence, a marked difference from the contribution made to *S. cerevisiae*.

Although *S. cerevisiae* do not form hyphae for the invasion of host tissues, filaments are formed during other biological functions. In *S. cerevisiae*, the ability of *SPT20* mutant to form filaments is impaired [14], [26], [44]–[46]. Combined with our results, the data strongly indicates that *SPT20* acts as an activator of filamentous and biofilm growth. It may affect the expression of hyphal-specific genes. In addition to *SPT20*, several SAGA related genes such as *SPT6*, *ADA2*, *SPT3* have shown to be related for the filamentous growth of *C. albicans*. The genes *SPT6* and *ADA2* play a positive role in filamentous growth [18], [47], while *SPT3* plays a negative role [26]. Both *ADA2* and *SPT3* play an important role in pathogenicity [18], [26].

In this work, we show that *C. albicans SPT20* is associated with virulence. It is likely that the defects in hyphae and biofilm formation of the *spt20Δ/Δ* mutant and the sensitivity to cell wall and osmotic stresses may explain the loss of virulence in both invertebrates and mammals. Moreover, *SPT20* plays a vital role in cell morphology and distribution of nuclear material, which may lead to the defects in filamentation and biofilm formation directly when this gene is deleted.

## Supporting Information

Figure S1 **The expression of** ***CHT3*** **was not affected by** ***spt20Δ/Δ*** **.** *CHT3* mRNA levels were tested by RT-PCR in wild-type strain and *spt20Δ/Δ* strain. No significant difference between the two strains was found(P>0.05).
(TIF)

Table S1 **Primers for the disruption and reconstitution of** ***SPT20*** **.**
(DOCX)

Table S2 **Primers used to identify replacements of the** ***SPT20*** **allels.**
(DOCX)
