# Supplementary figures and images for: The Role of Candida albicans SPT20 in Filamentation, Biofilm Formation and Pathogenesis
Source: PLoS One. 2014 Apr 14;9(4):e94468. doi: 10.1371/journal.pone.0094468 (PMC3986095; doi:10.1371/journal.pone.0094468)

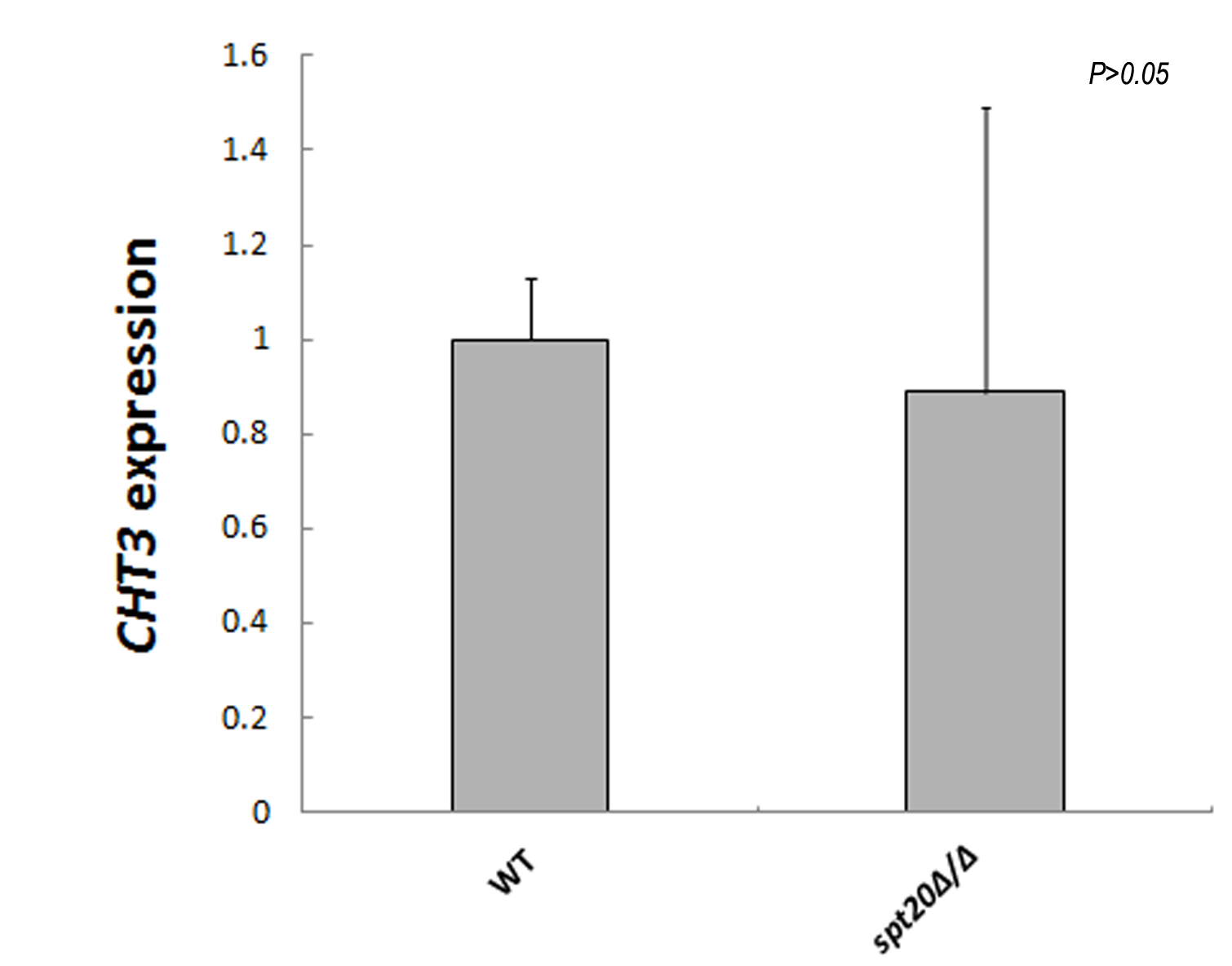

Supplement: Figure S1 — The expression of CHT3 was not affected by spt20Δ/Δ . CHT3 mRNA levels were tested by RT-PCR in wild-type strain and spt20Δ/Δ strain. No significant difference between the two strains was found(P>0.05). (TIF) [file pone.0094468.s001.tif]
