# Supplementary material for: The Role of Candida albicans SPT20 in Filamentation, Biofilm Formation and Pathogenesis
Source: PLoS One. 2014 Apr 14;9(4):e94468. doi: 10.1371/journal.pone.0094468 (PMC3986095; doi:10.1371/journal.pone.0094468)
Supplement: Table S1 — Primers for the disruption and reconstitution of SPT20 . (DOCX) [file pone.0094468.s002.docx]

**Table S1** Primers for the disruption and reconstitution of *SPT20*

|  | *Name* | *Sequence* |
| --- | --- | --- |
| Disruption | SPT20-up-FWD | ccc**gggccc** GAAACTGAAGAGTGGGTGAAGTCAG |
|  | SPT20-up-RV | ccc**ctcgag** TGCTGCTGCTGCTGCTGCTGAC |
|  | SPT20-down-FWD | ccc**ccgcgg** GCCGCTCCCCAAACTCAACAA |
|  | SPT20-down-RV | ccc**gagctc** GCAACAATTGCAGCACTTGGAATTT |
| Reconstitution | SPT20-FWD | ccc**gggccc** GAAACTGAAGAGTGGGTGAAGTCAG |
|  | SPT20-RV | cccctcgag GCAACAATTGCAGCACTTGGAATTT |
