# Supplementary material for: The Role of Candida albicans SPT20 in Filamentation, Biofilm Formation and Pathogenesis
Source: PLoS One. 2014 Apr 14;9(4):e94468. doi: 10.1371/journal.pone.0094468 (PMC3986095; doi:10.1371/journal.pone.0094468)
Supplement: Table S2 — Primers used to identify replacements of the SPT20 allels. (DOCX) [file pone.0094468.s003.docx]

**Table S2** Primers used to identify replacements of the *SPT20* allels

| *Name* | *Sequence* |
| --- | --- |
| 1 Ident F | GACTCGGCACTCCATCGATTC |
| 2 Ident R | CTGTTGTAAGTTCTTCGCTTGT |
| 3 Casset F | GCTTTCGGTCGCTGTTCTCA |
| 4 Casset R | TGTTAGGCGTCATCCTGTGC |
| 5 Up-F | GCCGATATGACCCTGCTATTA |
| 6 Up-R | CGTGTCTTCGTCTATCGCCT |
| 7 Dn-F | TCTCGGCGGCATTGACCTCTT |
| 8 Dn-R | GCAAGTACTGAGTCCAAGTGG |
